# Liver failure secondary to alcoholic liver disease carries a worse prognosis than other aetiologies of liver failure: retrospective analysis of routine biochemical markers in critically ill patients with liver failure

**DOI:** 10.1186/cc10998

**Published:** 2012-03-20

**Authors:** E McCarron, I Welters

**Affiliations:** 1 Royal Liverpool University Hospital, Liverpool, UK

## Introduction

Patients with liver failure in the critical care unit frequently provide physicians with problems about management and prognosis. Alcoholic liver disease (ALD) in particular is showing an increase in admission and mortality in the UK [1]. Current biochemical tests make it difficult to differentiate between types and severity of liver damage and fail to give a true idea about prognosis and outcome, often only showing low-grade derangements before hyperacute decompensation of liver function. The aim of this study was to look at various liver function tests (LFTs) routinely recorded in patients admitted to critical care with liver failure, to see whether they differed between ALD and nonalcoholic aetiologies (NALD); that is, drug overdose and nonalcoholic steatohepatitis, and so forth. We also aimed to assess their prognostication value and relation to severity of disease scores.

## Methods

A total of 119 patients admitted to the ITU with liver failure (66 ALD and 53 NALD) between 2008 and 20011 were included. Each patient had admitting electrolytes, haematology, LFTs and clotting studies along with APACHE II score, length of stay and ventilation and vital organ support requirement.

## Results

ALD patients were found to have lower sodium (mean 135.56; *P *= 0.004) and be hypocalcaemic (*P *= 0.015), as well as having more deranged LFTs (*P *< 0.001) and clotting studies (*P *< 0.001). ALD patients also had longer ITU stays (*P *< 0.001) and higher mortality rates (45.45% ALD vs. 13.2% NALD). Receiver-operated curve analysis revealed that current biochemical markers (ALT, PT, GGT, albumin) are not sensitive and specific enough in detecting ALD. The prothrombin time yielded the best area under the curve with 80.4% in ALD versus 71.7% in NALD. None of the markers was discriminatory for determining the type of liver damage.

## Conclusion

Our results suggest that currently used markers of liver disease are neither sensitive nor specific enough in patients with failure secondary to ALD. Research is needed to develop novel biomarkers to better prognosticate outcome. Aetiology of acute-on-chronic liver failure plays a major role in determining outcome, and subgroups of liver patients should be analysed individually. Studies [2,3] have shown that various markers are released depending on the type of damage and differ in acute liver damage of different origin. Better understanding of their role could prove useful in these patients.
